# Acute Severe Hypovolemic Hyponatremia in a Patient on Intravenous Dexamethasone

**DOI:** 10.7759/cureus.23080

**Published:** 2022-03-11

**Authors:** Sameer Peer, Dinesh A Sharma, Chandrajit Prasad, Karthik K

**Affiliations:** 1 Neuroimaging and Interventional Radiology, National Institute of Mental Health and Neurosciences, Bangalore, IND

**Keywords:** accidental head trauma, carotico-cavernous fistula, renin-angiotensin-aldosterone system, acute hyponatremia, dexamethasone

## Abstract

Hyponatremia is a commonly encountered electrolyte imbalance with varied etiology. Hyponatremia can be broadly classified as hypotonic, isotonic, and hypertonic hyponatremia based on the tonicity of plasma. Hypotonic hypovolemia is further classified as hypovolemic, euvolemic, and hypervolemic hyponatremia based on the volume status. Gastrointestinal fluid and electrolyte losses, secondary to vomiting and diarrhea, is an important predisposition to hypotonic hypovolemic hyponatremia. The renin-angiotensin-aldosterone system (RAAS) and antidiuretic hormone (ADH) play a pivotal role in maintaining intravascular volume and serum sodium concentration. Dexamethasone is a potent glucocorticoid with minimal mineralocorticoid activity. It negatively affects the hypothalamic-pituitary-adrenal axis and the renin-angiotensin-aldosterone system, particularly with prolonged administration. In the index case, acute severe hypovolemic hyponatremia ensued on the third post-procedure (endovascular embolization of traumatic carotico-cavernous fistula (CCF)) day while the patient was on intravenous dexamethasone. This case underscores that even small fluid and electrolyte imbalance in the setting of dexamethasone therapy may lead to severe hypovolemic hyponatremia, which requires specific therapy.

## Introduction

Hyponatremia is defined as a serum sodium concentration of less than 135 mmol/L [1]. A serum sodium level of ≤120 mmol/L is generally regarded as severe hyponatremia [2]. Temporal evolution of the stated condition within 48 hours constitutes acute hyponatremia [3]. Acute hyponatremia is a medical emergency and manifests with confusion, seizures, brain edema, brain herniation, coma, and death [3]. In a given case, assessment of fluid volume status is critical in the management of hyponatremia [3-5]. The renin-angiotensin-aldosterone system (RAAS) in conjunction with antidiuretic hormone (ADH) plays a pivotal role in the maintenance of sodium and fluid levels in the body. In a scenario of acute volume/fluid loss, restorative homeostasis is achieved through the activation of the renin-angiotensin-aldosterone system and the release of ADH from the posterior pituitary, which causes reduced renal excretion of sodium and water [6]. Dexamethasone, an anti-inflammatory pharmacological agent, is a potent steroid with a predominant glucocorticoid activity and a notably insignificant mineralocorticoid activity [7]. Dexamethasone-induced hypothalamic-pituitary-adrenal axis suppression cascades to reduced adreno-corticotrophic hormone (ACTH) and serum cortisol level. Abrupt withdrawal of dexamethasone after prolonged therapy may precipitate acute adrenal insufficiency [8]. Besides, dexamethasone also suppresses aldosterone release from the adrenal cortex [9,10]. In a setting of acute volume depletion, it is not inconceivable that dexamethasone may precipitate hypovolemic hyponatremia attributable to its limited mineralocorticoid activity. We present a case of acute severe hyponatremia in a 39-year-old female who underwent therapeutic embolization of traumatic direct carotico-cavernous fistula (CCF) followed by intravenous dexamethasone therapy. The patient developed acute hyponatremia following two bouts of vomiting and was managed to her recovery with normal saline infusion. This case underscores the pathophysiological mechanism that may lead to acute hyponatremia in patients on intravenous dexamethasone after relatively mild volume depletion. The treating physician should be aware of such a possibility, which may be challenging to recognize otherwise.

## Case presentation

**Figure 1:**
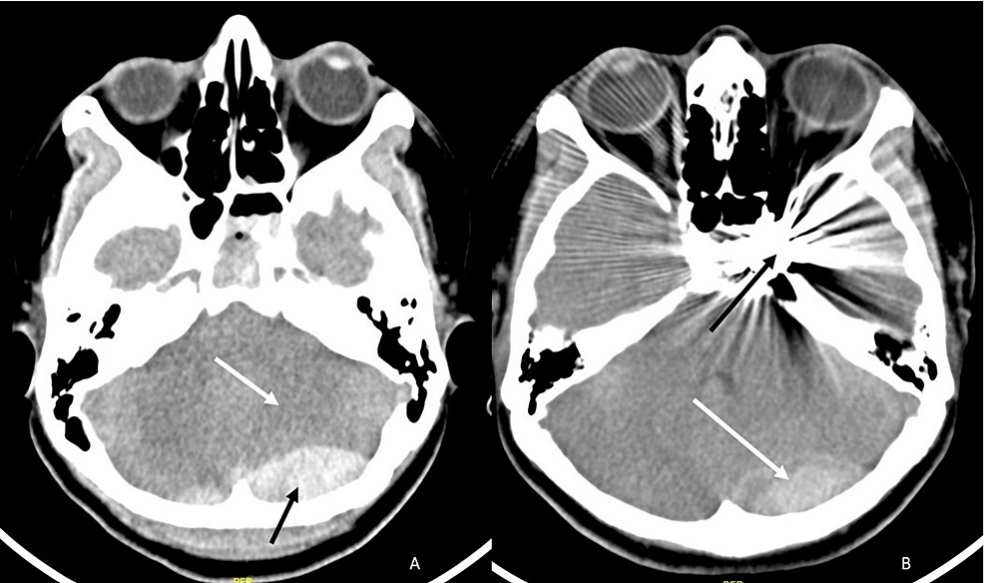
Computed tomography of the brain (A) Acute extradural hematoma is noted in the left occipital region (black arrow) with a mass effect on the left cerebellar hemisphere (white arrow). (B) Post-embolization of the carotico-cavernous fistula shows streak artifacts due to coil mass and embosylate cast (black arrow). The extradural hematoma (white arrow) has not increased in size.

**Figure 2:**
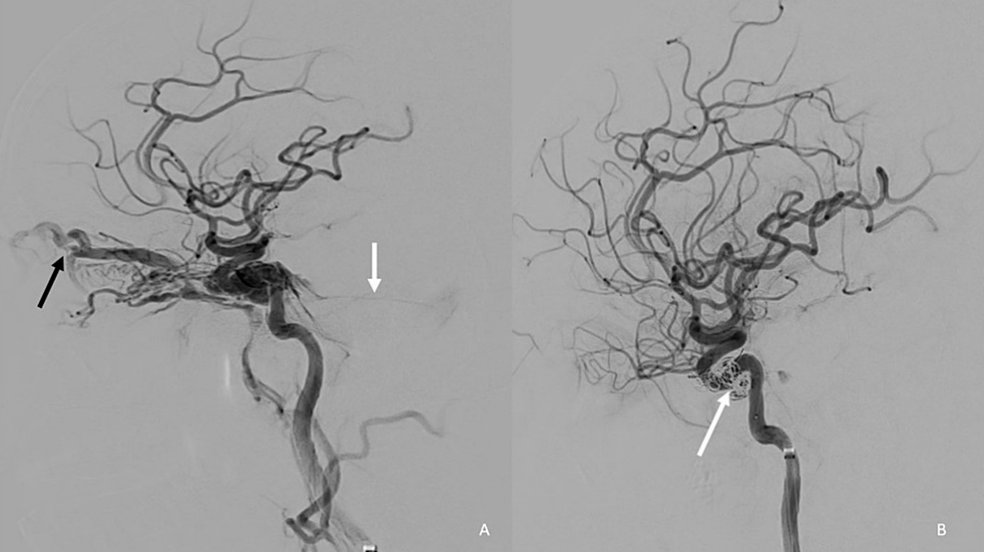
Digital subtraction angiogram (A) Lateral view of the digital subtraction angiogram shows evidence of direct carotico-cavernous fistula with reflux of contract into the superior ophthalmic vein (black arrow) and into the superior petrosal vein (white arrow). (B) Post-embolization of carotico-cavernous fistula. Coil mass and embosylate cast noted in the cavernous sinus (black arrows). Note that there is no residual filling of the fistula, superior ophthalmic vein, or superior petrosal sinus.

A 39-year-old female presented with left-sided proptosis of three days following a road traffic accident. Following trauma, the patient developed left CN III and CN IV palsy resulting in left-sided external ophthalmoplegia. The left pupil was dilated. Direct and consensual light reflexes were not demonstrated on the left side. Also, the left pupil did not show reactivity on the performance of accommodation reflex. Pulsatile bruit was discernible over the left globe. A clinical picture corollary to history was consistent with the diagnosis of traumatic carotico-cavernous fistula (CCF). Computed tomography (CT) evaluation of the brain showed an acute extradural hematoma in the left occipital region with a mass effect on the cerebellum and a left cavernous sinus prominence (Figure 1A). Digital subtraction angiogram (DSA) demonstrated a left direct carotico-cavernous fistula (Figure 2A), which was therapeutically embolized with coils and liquid embolics. Post-procedure, complete obliteration of the fistula was achieved (Figure 2B). Post-procedure CT demonstrated no interval change in the size of the hematoma and was managed conservatively (Figure 1B).

In view of cranial nerve palsies and per our institutional protocol, intravenous dexamethasone (4 mg IV q6h) was initiated to mitigate the inflammation secondary to CCF’s thrombosis. On the second post-procedure day, the patient had two bouts of vomiting of about 150-200 mL of vomitus. In the ensuing 2-3 hours, her sensorium worsened, and she developed drowsiness and confusion. She was unable to obey any commands and was not vocalizing. Serum electrolyte analysis revealed a serum sodium level of 114.7 mmol/L confirming the diagnosis of acute severe hyponatremia. Acute encephalopathy with concurrent hyponatremia warranted intervention, the serum sodium was alarmingly low, and intravenous infusion of 3% saline was initiated at the rate of 1.5 mL/kg/hour. Toward tailoring appropriate management, serum osmolality, urine osmolality, and urine sodium levels were measured, as shown in Table 1.

**Table 1:** Summary of laboratory parameters for the diagnosis of hypovolemic hyponatremia and posttreatment changes

Laboratory parameters	At the time of admission	Day 2 post-procedure	At the time of discharge (five days post-procedure)	Normal reference range
Serum sodium (mmol/L)	136.7	114.7	136.2	136–145
Serum potassium (mmol/L)	4.45	3.48	4.28	3.5–5
Serum chloride (mmol/L)	101.7	81.3	100.3	98–107
Serum urea (mg/dL)	18	17	18	16.6–48.5
Serum creatinine (mg/dL)	0.7	0.8	0.7	0.5–0.9
Serum osmolality (mOsm/kg)	-	260	282	275–295
24-hour urine osmolality (mOsm/kg)	-	130	214	300–900
Urine sodium (mmol/L)	-	36.2	122.10	40–220
Random blood glucose (mg/dL)	138	121	130	70–140

Serum osmolality, urine sodium, and urine osmolality were 260 mOsm/kg, 36.2 mmol/L, and 130 mOsm/kg, respectively. Taking into consideration the overall clinical picture and the pattern of electrolyte imbalance, a diagnosis of hypovolemic hyponatremia was established. Repeat serum sodium level after six hours revealed a serum sodium level of 123.8 mmol/L. As rapid correction of hyponatremia predisposes to osmotic demyelination, 3% saline infusion was replaced with 0.9% saline, which was infused at a rate of 1.5 mL/kg/hour. Three days later, her serum sodium levels and serum osmolality were normalized. Dexamethasone was tapered and stopped two days later. The patient’s clinical status and sensorium improved, and she was subsequently discharged in a stable condition.

## Discussion

Dexamethasone is a synthetic steroid with a predominant glucocorticoid activity with a longer duration of action and is more potent than hydrocortisone and prednisone [11,12]. Dexamethasone suppresses the hypothalamic-pituitary-adrenal axis, a characteristic exploited in diagnosing Cushing’s syndrome (dexamethasone suppression test) [13]. Prolonged administration of dexamethasone induces adrenal cortical atrophy, and abrupt discontinuation following protracted use may precipitate acute adrenal insufficiency [8]. Additionally, dexamethasone suppresses the release of aldosterone from the adrenal cortex [9,10].

Physiologically, sodium and water loss stimulates the juxtaglomerular apparatus to secrete renin [14]. Renin is an enzyme that converts angiotensinogen to angiotensin I. Angiotensin I is converted to angiotensin II by angiotensin-converting enzyme 2 (ACE2), which is predominantly expressed in the lungs. Angiotensin II acts at the level of renal glomeruli to constrict afferent and efferent arterioles and also triggers aldosterone release from the adrenal cortex [15,16]. Aldosterone exerts an influence on the renal tubules, which leads to increased sodium and water resorption with concomitant potassium excretion [14-16]. In short, the renin-angiotensin-aldosterone axis is critical in maintaining the electrolyte milieu through multiple pathways. In conjunction with the RAAS pathway, reduced plasma volume and sodium loss stimulate the secretion of ADH, which acts on the distal renal tubular system to increase water resorption, thereby helping in the restoration of the plasma volume [15]. Dexamethasone suppresses aldosterone release from the adrenal cortex through a negative feedback mechanism on adreno-corticotrophic hormone (ACTH) release [9,10]. The suppression of aldosterone release by dexamethasone impairs the renin-angiotensin-aldosterone pathway, thereby inhibiting the restorative actions on the sodium and fluid levels. Since dexamethasone exerts negligible mineralocorticoid activity, it engenders a risk of hypovolemic hyponatremia. This explains the acute severe hypovolemic hyponatremia caused by just two bouts of vomiting, leading to loss of approximately 150-200 mL of gastrointestinal fluid, which, otherwise, in a person with no known comorbid illness, would not lead to such a severe reduction of serum sodium levels.

Hyponatremia is etiologically diverse; a few relevant differentials were considered in our case. Syndrome of inappropriate antidiuretic hormone (SIADH) is known to occur following head trauma [17]. However, in SIADH, the urine osmolality remains high in conjunction with a urine sodium level of >40 mmol/L that remains uncorrected with normal saline infusion. However, this condition can be ameliorated through the proper use of hypertonic saline and fluid restriction [18]. Another confounder following head trauma is “cerebral salt wasting syndrome,” which is characterized by increased urinary sodium excretion due to elevated brain natriuretic peptide level and polyuria [19]. The possibility of pseudohyponatremia was excluded as the patient was euglycemic and did not have hyperlipidemia. A summary of the possible causes of hyponatremia is presented in Table 2.

**Table 2:** Summary of various causes of hyponatremia

Hypovolemic hyponatremia
Gastrointestinal losses (diarrhea and vomiting), third space fluid depletion (hypoalbuminemia, small bowel obstruction, and acute pancreatitis), osmotic diuresis (hyperglycemia and mannitol), diuretic agents, mineralocorticoid deficiency, cerebral salt wasting, salt-losing nephropathy
Euvolemic hyponatremia
Syndrome of inappropriate antidiuretic hormone (SIADH), Addison’s disease, psychogenic polydipsia (potomania), drug induced (desmopressin, oxytocin, selective serotonin reuptake inhibitors, tricyclic antidepressants, opioids, carbamazepine, vincristine, cyclophosphamide, chlorpropamide, nonsteroidal anti-inflammatory drugs, drugs of abuse such as methylenedioxymethamphetamine (MDMA) or ecstasy, etc.
Hypervolemic hyponatremia
Renal causes (acute renal failure, chronic renal failure, and nephrotic syndrome), cirrhosis, congestive cardiac failure, iatrogenic (medical and surgical procedures such as cardiac catheterization, colonoscopy, transurethral resection of the prostate (due to excessive fluid administration))

There are several reports iterating the therapeutic role of dexamethasone in hyponatremic conditions [20]; however, to the best of our knowledge, there is no report describing acute severe hyponatremia following dexamethasone therapy. Our case underscores the possible contribution of dexamethasone to the pathophysiology of acute hyponatremia in conditions predisposing to acute fluid and electrolyte loss.

## Conclusions

Dexamethasone exerts an inhibitory effect on the hypothalamic-pituitary-adrenal axis and aldosterone release from the adrenal cortex. Due to dexamethasone’s negligible mineralocorticoid activity, even relatively mild fluid and electrolyte loss in a patient receiving dexamethasone may predispose the patient to acute and severe hypovolemic hyponatremia. However, the condition is reversible if prompt fluid and sodium correction is instituted to prevent the development of acute cerebral edema.

## References

[1] Verbalis JG, Goldsmith SR, Greenberg A, Korzelius C, Schrier RW, Sterns RH, Thompson CJ (2013). Diagnosis, evaluation, and treatment of hyponatremia: expert panel recommendations. Am J Med.

[2] Sterns RH (2018). Treatment of severe hyponatremia. Clin J Am Soc Nephrol.

[3] Sahay M, Sahay R (2014). Hyponatremia: a practical approach. Indian J Endocrinol Metab.

[4] Babaliche P, Madnani S, Kamat S (2017). Clinical profile of patients admitted with hyponatremia in the medical intensive care unit. Indian J Crit Care Med.

[5] Reddy SN, Rangappa P, Jacob I, Janakiraman R, Rao K (2016). Efficacy of conivaptan and hypertonic (3%) saline in treating hyponatremia due to syndrome of inappropriate antidiuretic hormone in a tertiary Intensive Care Unit. Indian J Crit Care Med.

[6] Bermejo S, García CO, Rodríguez E (2018). The renin-angiotensin-aldosterone system blockade in patients with advanced diabetic kidney disease. Nefrologia (Engl Ed).

[7] Liu D, Ahmet A, Ward L (2013). A practical guide to the monitoring and management of the complications of systemic corticosteroid therapy. Allergy Asthma Clin Immunol.

[8] Broersen LH, Pereira AM, Jørgensen JO, Dekkers OM (2015). Adrenal insufficiency in corticosteroids use: systematic review and meta-analysis. J Clin Endocrinol Metab.

[9] Inoue K, Yamazaki Y, Kitamoto T (2018). Aldosterone suppression by dexamethasone in patients with KCNJ5-mutated aldosterone-producing adenoma. J Clin Endocrinol Metab.

[10] Litchfield WR, New MI, Coolidge C, Lifton RP, Dluhy RG (1997). Evaluation of the dexamethasone suppression test for the diagnosis of glucocorticoid-remediable aldosteronism. J Clin Endocrinol Metab.

[11] BU JJ, BL RL, LU L, PE RE, WH GD (1958). Studies on dexamethasone, a new synthetic steroid, in rheurheumatoid arthritis: a preliminary report; adrenal cortical, metabolic and early clinical effects. Arthritis Rheum.

[12] Meikle AW, Tyler FH (1977). Potency and duration of action of glucocorticoids. Effects of hydrocortisone, prednisone and Dexamethasone on human pituitary-adrenal function. Am J Med.

[13] Esfahanian F, Kazemi R (2010). Overnight dexamethasone suppression test in the diagnosis of Cushing's disease. Acta Med Iran.

[14] Muñoz-Durango N, Fuentes CA, Castillo AE, González-Gómez LM, Vecchiola A, Fardella CE, Kalergis AM (2016). Role of the renin-angiotensin-aldosterone system beyond blood pressure regulation: molecular and cellular mechanisms involved in end-organ damage during arterial hypertension. Int J Mol Sci.

[15] Kuba K, Imai Y, Penninger JM (2006). Angiotensin-converting enzyme 2 in lung diseases. Curr Opin Pharmacol.

[16] Sparks MA, Crowley SD, Gurley SB, Mirotsou M, Coffman TM (2014). Classical renin-angiotensin system in kidney physiology. Compr Physiol.

[17] Dick M, Catford SR, Kumareswaran K, Hamblin PS, Topliss DJ (2015). Persistent syndrome of inappropriate antidiuretic hormone secretion following traumatic brain injury. Endocrinol Diabetes Metab Case Rep.

[18] Runkle I, Villabona C, Navarro A, Pose A, Formiga F, Tejedor A, Poch E (2014). Treatment of hyponatremia induced by the syndrome of Inappropriate antidiuretic hormone secretion: a multidisciplinary spanish algorithm. Nefrologia.

[19] Heras Benito M, Iglesias P, Guevara P, Sánchez Hernández R, Fernández-Reyes MJ (2008). Hiponatremia secundaria a síndrome pierde sal cerebral asociado a meningitis bacteriana [Hyponatremia secondary to cerebral salt-wasting syndrome associated to bacterial meningitis]. Nefrologia.

[20] Kazama I, Tamada T, Nakajima T (2015). Hyponatremia due to secondary adrenal insufficiency successfully treated by dexamethasone with sodium chloride. Am J Case Rep.

